# Supplementary material for: The lactonase BxdA mediates metabolic specialisation of maize root bacteria to benzoxazinoids
Source: Nat Commun. 2024 Aug 2;15:6535. doi: 10.1038/s41467-024-49643-w (PMC11297187; doi:10.1038/s41467-024-49643-w)
Supplement: Supplementary file 1 — Supplementary Information [file 41467_2024_49643_MOESM1_ESM.pdf]

Supplementary Information

The lactonase BxdA mediates metabolic specialisation of maize root bacteria to benzoxazinoids

Lisa Thoenen[1,2], Marco Kreuzer[3], Christine Pestalozzi[2], Matilde Florean[4], Pierre Mateo[1], Tobias Züst[1,5], Anlun Wei[2], Caitlin Giroud[2], Liza Rouyer[6], Valentin Gfeller[1], Matheus D. Notter[7], Eva Knoch[6,8], Siegfried Hapfelmeier[7], Claude Becker[6,8], Niklas Schandry[6,8], Christelle A. M. Robert[1], Tobias G. Köllner[4], Rémy Bruggmann[3], Matthias Erb[1*], Klaus Schlaeppi[1, 2*]

[1] Institute of Plant Sciences, University of Bern, Bern, Switzerland

[2] Department of Environmental Sciences, University of Basel, Basel, Switzerland

[3] Interfaculty Bioinformatics Unit, University of Bern, Bern, Switzerland

[4] Department of Natural Product Biosynthesis, Max Planck Institute for Chemical Ecology, Jena, Germany

[5] Department of Systematic and Evolutionary Botany, University of Zurich, Zurich, Switzerland

[6] LMU Biocenter, Faculty of Biology, Ludwig-Maximilians-University Munich, Martinsried, Germany

[7] Institute for Infectious Diseases, University of Bern, Bern, Switzerland

[8] Gregor Mendel Institute of Molecular Plant Biology GmbH, Austrian Academy of Sciences, Vienna BioCenter (VBC), Vienna, Austria

Index

- Supplementary Results
- Supplementary Figures
- Supplementary Tables
- References

Supplementary Results

Screening of Arabidopsis bacteria for AMPO formation

Complementary to screening the MRB strains for AMPO formation, we also screened some Arabidopsis bacteria for this trait. Of the AtSphere collection[1], strains with high 16S rRNA gene sequence similarity to the strains of the MRB collection were selected. This resulted in a selection of 57 strains that were isolated either from Arabidopsis roots or leaves, or the soil where Arabidopsis was grown to isolate root strains of the AtSphere collection. Goal was not a systematic comparison of the two collections (which would not make sense with the varying compartments of isolation) but to expand the number of strains (i.e., pairs of genomes and AMPO formation phenotypes) for subsequent comparative genomics. We plated the 57 Arabidopsis strains on MBOA-containing agar plates and classified their colony and surrounding media colour after 10 days incubation time as for the MRB collection. Supplementary Fig. 3 illustrates the classes non (no colour change compared to DMSO control plates), weak (light red colouration) or strong AMPO-formers (dark red colour). We found four strong AMPO-formers among the 57 tested strains (Supplementary Fig. 7a). These four strong AMPO-formers were *Acinetobacter* Root1280 and Leaf130, *Xanthomonas* Leaf131 and *Achromobacter* Root83. However, compared to strong AMPO-formers of maize, the Arabidopsis strains did not efficiently degrade MBOA in liquid culture (Supplementary Fig. 7b). Most importantly, this screen showed that none of the tested *Microbacterium* strains from Arabidopsis could form AMPO, which is in contrast to the microbacteria of maize with both AMPO-forming and non-AMPO-forming strains (Fig. 1d).

BxdA also degrades BOA

Maize primarily synthesizes methoxylated BXs with the chemical conversion pathway from exuded DIMBOA-Glc and DIMBOA to accumulating MBOA and AMPO in the rhizosphere[2] (Supplementary Fig. 1). Exudation of DIBOA-Glc and DIBOA and accumulation of BOA and APO is the analogous 'sequence' of BX degradation for plants like rye, which mainly produces non-methoxylated analogs. Wheat produces a mixture of both methoxylated and non-methoxylated BXs. The focus of this study was bacterial metabolisation of MBOA and AMPO formation (both methoxylated compounds) and complementary, we also tested the key observations with non-methoxylated BOA and eventual APO formation. Specifically, we tested (i) whether strong MBOA degrading *Microbacterium* and *Sphingobium* strains would also degrade BOA, (ii) in vitro activity of purified BxdA to degrade BOA and form APO and (iii) in vivo function of BxdA comparing BOA degradation of *Sphingobium* LSP13 and the mutant Δ3bxdA.

First, we selected the same set of strains as for kinetic characterisation of MBOA degradation and AMPO formation (Fig. 2c). We grew them for 68 h in 50% TSB with either 500

μM MBOA or BOA and analyzed the BX metabolites. Consistent with the earlier assays, *Sphingobium* LSP13, *Pseudoarthrobacter* LMD1 and *Microbacterium* LMB2 largely degraded the initial levels of MBOA and AMPO was formed in these cultures, while this was not observed for the other strains (Supplementary Fig. 11b). Similar phenotypes were observed when exposing these strains to BOA, with the strong MBOA-degraders also degrading BOA and forming small amounts of APO.

Next, we tested whether the purified BxdA protein would also degrade BOA and form APO in vitro. Compared to the empty vector control, we measured reduced BOA concentrations and low levels of APO in presence of BxdA (Supplementary Fig. 11c). In comparison with MBOA, far less BOA was degraded during the same incubation time and using the same assay settings (same protein amounts and same initial MBOA and BOA concentrations). This indicates that BxdA is less efficient in converting BOA to APO than converting MBOA to AMPO under the tested conditions. Hence, BxdA protein has some in vitro activity to metabolise non-metoxylated BOA to APO, albeit lower compared to their methoxylated analogs MBOA and AMPO.

Third, to study the in vivo function of BxdA, we examined both metabolisation of BOA and growth with BOA as sole carbon source in minimal medium comparing *Sphingobium* LSP13 wild-type and the Δ3bxdA mutant. While LSP13 grew in minimal media with MBOA or BOA as sole carbon sources, the mutant Δ3bxdA was severely impaired in growth with both carbon sources (Supplementary Fig. 11a). Metabolite analyses of these cultures revealed that LSP13 degraded MBOA and formed AMPO (Fig. 5c) as well as degraded BOA and formed small amounts of APO (Supplementary Fig. 11d). In contrast, the mutant Δ3bxdA failed to degrade both MBOA and BOA.

Taken together, these experiments revealed that the key conclusions of BxdA related to MBOA degradation and AMPO formation including its in vitro activity and in vivo function can be transferred to the degradation of non-methoxylated BOA and subsequent APO formation.

Supplementary Figures

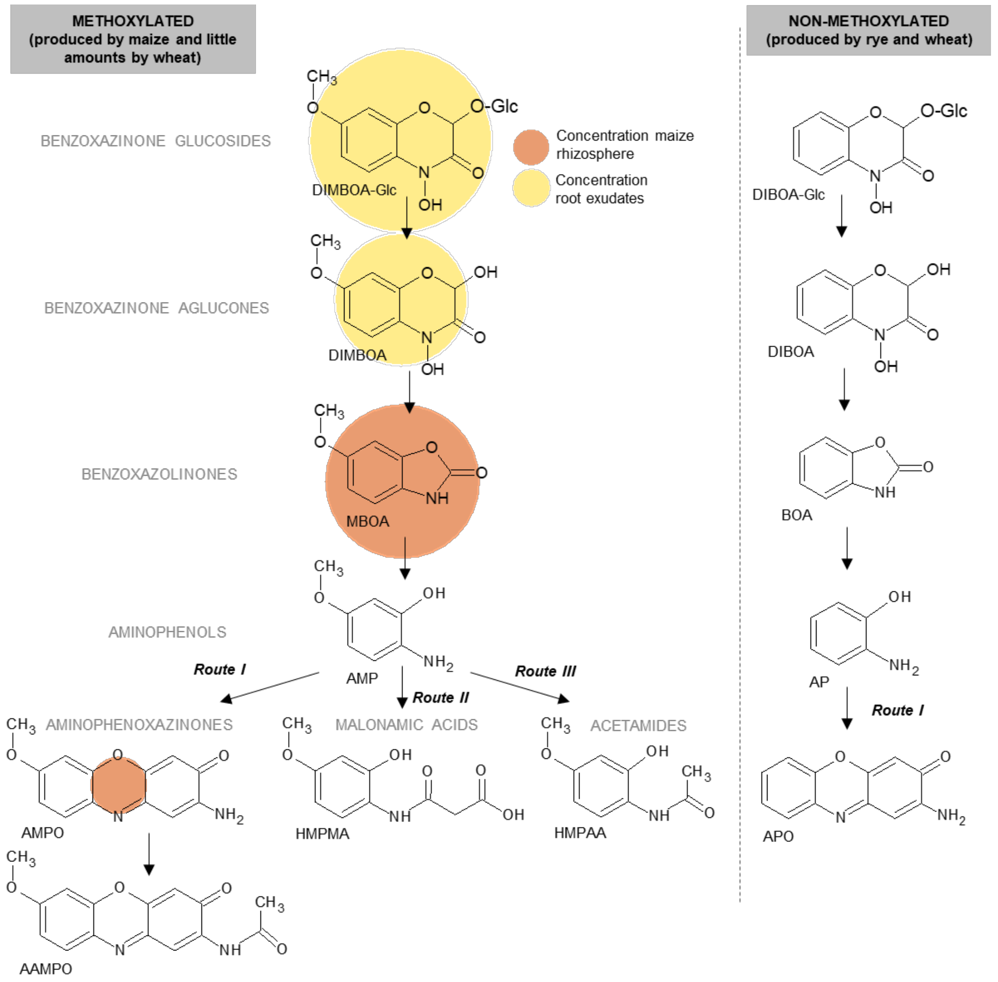

Abbreviation	Full name	Class	Mass [g/mol]	Formula	Rhizosphere [µg/kg FW]	Root exudates [µg/cm³]
DIMBOA-Glc	2-O-β-D-glucopyranosyl-2,4-dihydroxy-7-methoxy-(2H)-1,4-benzoxazin-3(4H)-one	Benzoxazinone glucoside	373.31	C15H19NO10	ND	0.153± 0.029
DIBOA-Glc	2-O-β-d-glucopyranosyl-4-hydroxy-(2H)-1,4-benzoxazin-3(4H)-one	Benzoxazinone glucoside	343.29	C14H17NO9	ND	ND
DIMBOA	2,4-dihydroxy-7-methoxy-1,4-benzoxazin-3-one	Benzoxazinone aglucone	211.17	C9H9NO5	0.93± 0.30	0.081± 0.026
DIBOA	2,4-dihydroxy-1,4-benzoxazin-3-one	Benzoxazinone aglucone	181.15	C8H7NO4	ND	ND
MBOA	6-methoxybenzoxazolin-2(3H)-one	Benzoxazolinone	165.15	C8H7NO3	1.9± 0.5	0.0030± 0.0007
BOA	Benzoxazolin-2-(3H)-one	Benzoxazolinone	135.10	C7H5NO2	ND	ND
AMP	2-amino-5-methoxyphenol	Aminophenol	139.15	C7H9NO2	ND	ND
AP	2-aminophenol	Aminophenol	109.13	C6H7NO	ND	ND
HMPAA	N-(2-hydroxy-4-methoxyphenyl)acetamide	Acetamide	181.19	C9H11NO3	ND	ND
HMPMA	N-(2-hydroxy-4methoxyphenyl)malonamic acid	Malonamic acid	225.2	C10H11NO5	ND	ND
AMPO	2-amino-7-methoxy-phenoxazin-3-one	Aminophenoxazinones	242.23	C13H10N2O3	0.13± 0.03	ND
APO	2-amino-(3H)-phenoxazin-3-one	Aminophenoxazinones	212.20	C12H8N2O2	ND	ND
AAMPO	2-acetylamino-7-methoxy-phenoxazin-3-one	Aminophenoxazinones	284.27	C15H12N2O4	ND	ND
AAPO	2-acetylamino-(3H)-phenoxazin-3-one	Aminophenoxazinones	254.24	C14H10N2O3	ND	ND

Supplementary Figure 1: Benzoxazinoid metabolites produced by plants and degradation pathways in soil. Bubble size represents the concentration of the compounds measured in the rhizosphere (orange) and root exudates (yellow) of maize. The table lists the full chemical name, the compound class, the molar mass, the chemical formula, and the concentrations in the rhizosphere (per kg FW, FW = fresh weight) and in root exudates (measured per root surface cm^3). Concentrations (mean ± standard deviation) are based on the measurements from Hu et al. 2018[3], ND = not detected.

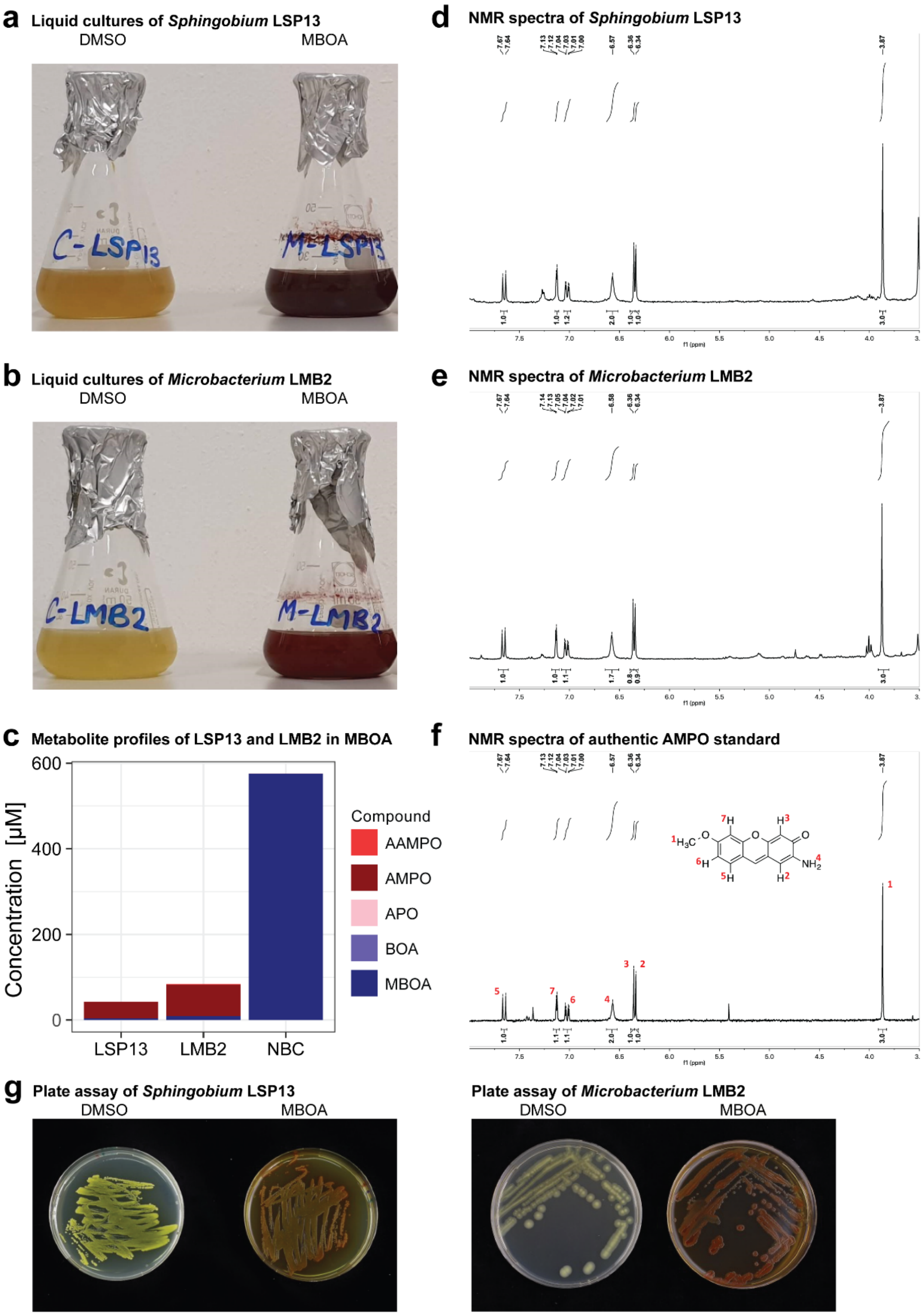

Supplementary Figure 2: AMPO phenotype and confirmation of AMPO formation by NMR. **a)** Pictures of liquid cultures of AMPO-forming strains *Sphingobium* LSP13 and **b)** *Microbacterium* LMB2 cultivated in 50% TSB supplemented with DMSO (left) or 500 μM MBOA (right). **c)** Metabolite profiles of LSP13 and LMB2 cultures grown in presence of MBOA for 68 h alongside the no bacteria control (NBC, n=1). **d)** NMR spectra of the red precipitate purified from LSP13 and **e)** LMB2 cultures grown in presence of MBOA for 68 h, and **f)** a pure AMPO sample. The pattern of peaks in the red precipitate extracted from bacterial cultures matches pure AMPO. **g)** Pictures of AMPO-forming strains *Sphingobium* LSP13 (left) and *Microbacterium* LMB2 (right) cultivated on 100% TSA plates containing DMSO (2 mL/L) or MBOA (200 mg/L; ~1’200 μM) for 10 days.

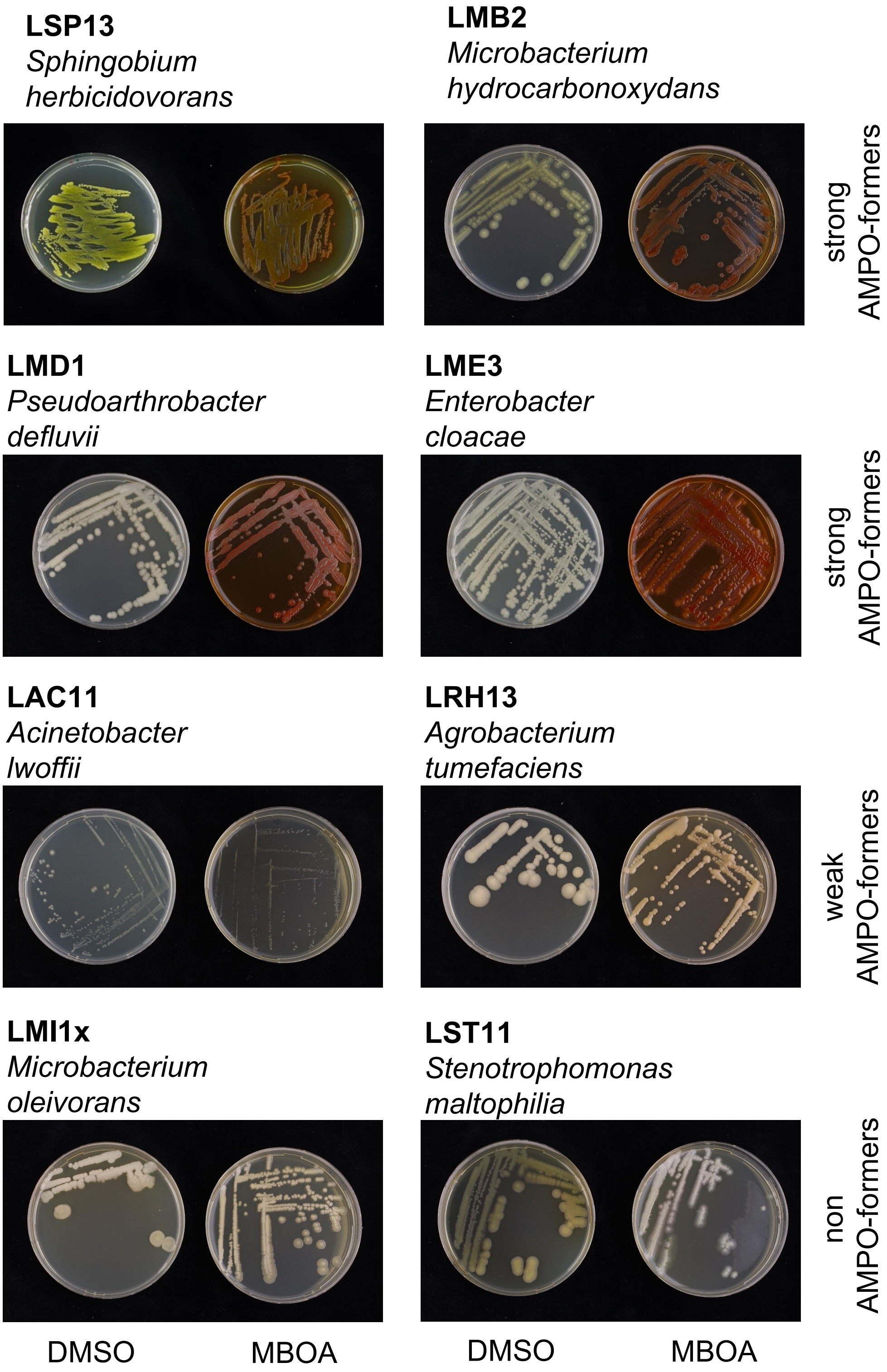

Supplementary Figure 3: Screening for AMPO formation on MBOA containing plates. Selected maize root bacteria on 100% TSA plates containing DMSO (2 mL/L; left) or MBOA (200 mg/L; ~1'200 µM; right) photographed after 10 days of incubation. Strong AMPO-formers provoke a clear (dark) red colour on MBOA plates while weak AMPO-formers show a pale red colouration. Non AMPO-formers do not show a colour change to light or dark red on MBOA compared to the control plates. Note that images of LPS13 and LMB2 are depicted in Supplementary Fig. 2 and shown again for comparison.

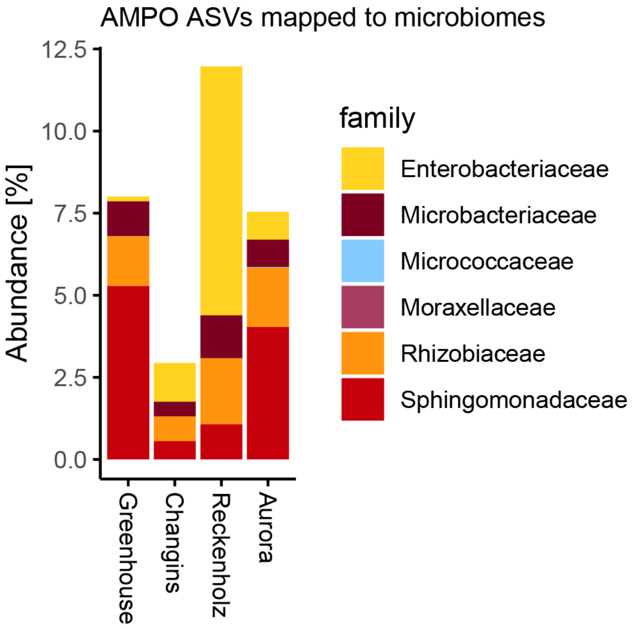

Supplementary Figure 4: Mapping of AMPO-forming maize root bacteria to microbiota data indicates that they are abundant on roots of BX-producing maize plants. The 16S rRNA gene sequences of the AMPO-forming maize strains were mapped to two maize root microbiota datasets. First, they were mapped to the greenhouse experiment described previously[3], of which most MRB strains were isolated from the roots of these wild-type B73 plants. Second, they were mapped to field data[4], where we reported bacterial communities on roots of wild-type B73 and W22 plants grown at the following locations: Changins and Reckenholz in Switzerland and Aurora in the US. The graph reports the cumulative relative abundances of all ASVs to which the sequences of the AMPO-forming isolates mapped to with at least 99% similarity.

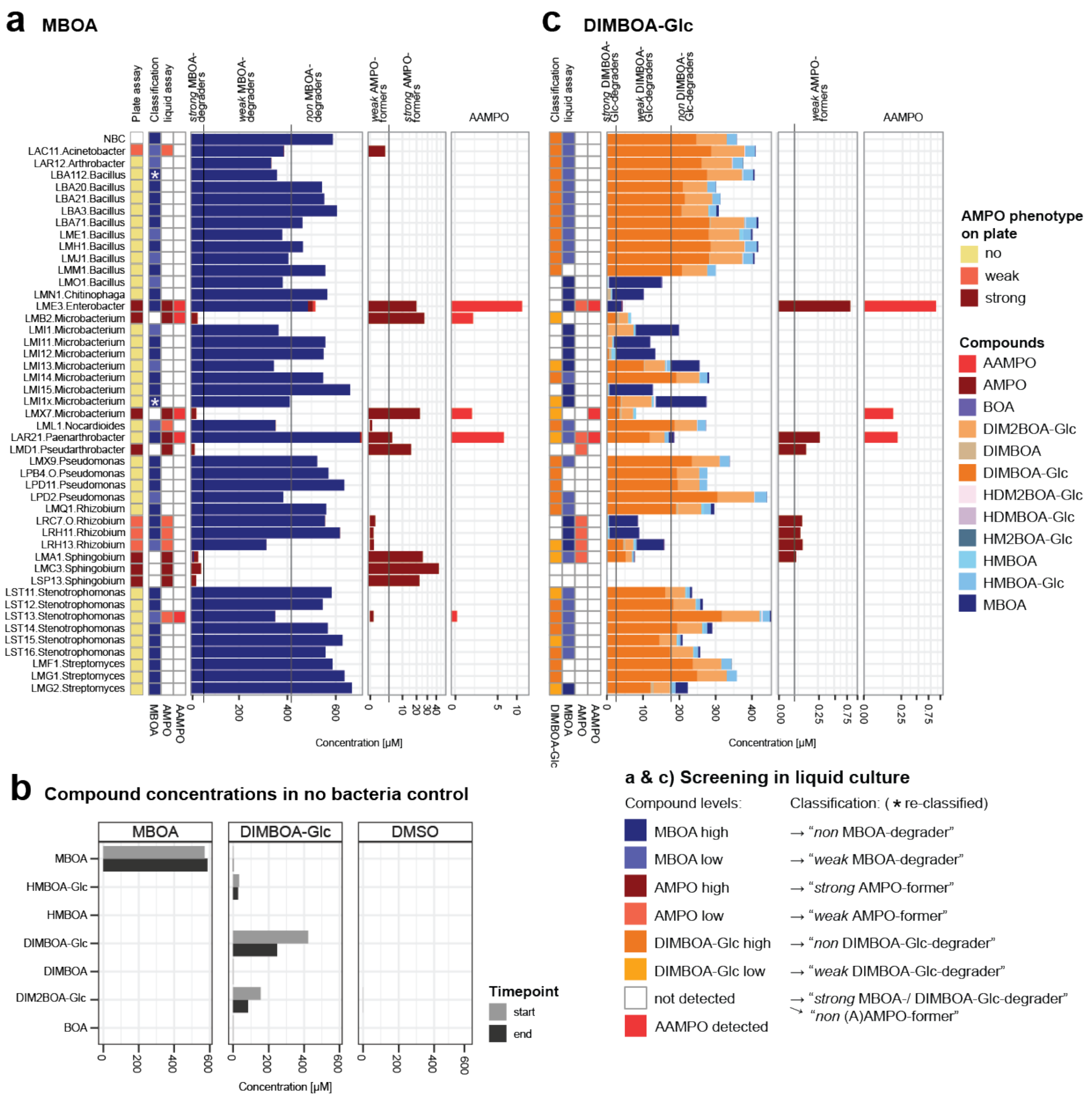

Supplementary Figure 5: Metabolisation of benzoxazinoids by maize root bacteria. We grew a set of 46 strains of the MRB collection in liquid cultures for 68 h in 50% TSB, either in presence of 500 µM MBOA or DIMBOA-Glc and quantified their metabolisation using UPLC-MS. The metabolite measurements (n=1) were made from three independently grown samples which were pooled in equal ratios before analysis. Goals were to chemically validate AMPO formation and to test whether maize root bacteria degrade MBOA or DIMBOA-Glc without forming AMPO. **a)** Reports the assay with 500 µM MBOA. For reference, the first panel reports the AMPO formation phenotypes as tested with MBOA-containing plates (data subset from Figure 1d). The second panel with three columns shows the qualitative classifications of the strains for MBOA degradation (non, weak or strong MBOA-degraders) and AMPO formation (non, weak or strong AMPO-formers). These classifications are based on the metabolisation profiles of all detected degradation products (third panel) represented in stacked bar graphs. The fourth and fifth panel repeats the data for AMPO and AAMPO (not used for classifications because of very low levels) with suitable scales. The vertical lines in the metabolisation profiles refer to the cut-offs used for classification (see methods for definitions). The asterisk (*) marks the re-classification of the strains LBA112 and LMI1x to 'non MBOA-degraders' because two additional experiments (Fig. 2c, Supplementary Fig. 11b) revealed that they do not degrade MBOA. **b)** The same assay was also performed with 500 µM DIMBOA-Glc, which is not commercially available and was therefore purified from maize (~70% purity). We measured compound concentrations in the no bacteria control (NBC) samples at the start and the end of the MBOA and DIMBOA-Glc experiments and document here other co-purified benzoxazinoids (n=1). **c)** Reports the assay with DIMBOA-Glc with the qualitative classifications of the strains for DIMBOA-Glc degradation (non, weak or strong DIMBOA-Glc-degraders), MBOA and AMPO formation (non, weak or strong MBOA-/AMPO-formers) in the first panel. The corresponding metabolisation profiles of all detected degradation products and the close ups for AMPO and AAMPO are in the second, third and fourth panels, respectively. The vertical lines refer to the cut-offs used for classification (see methods for definitions).

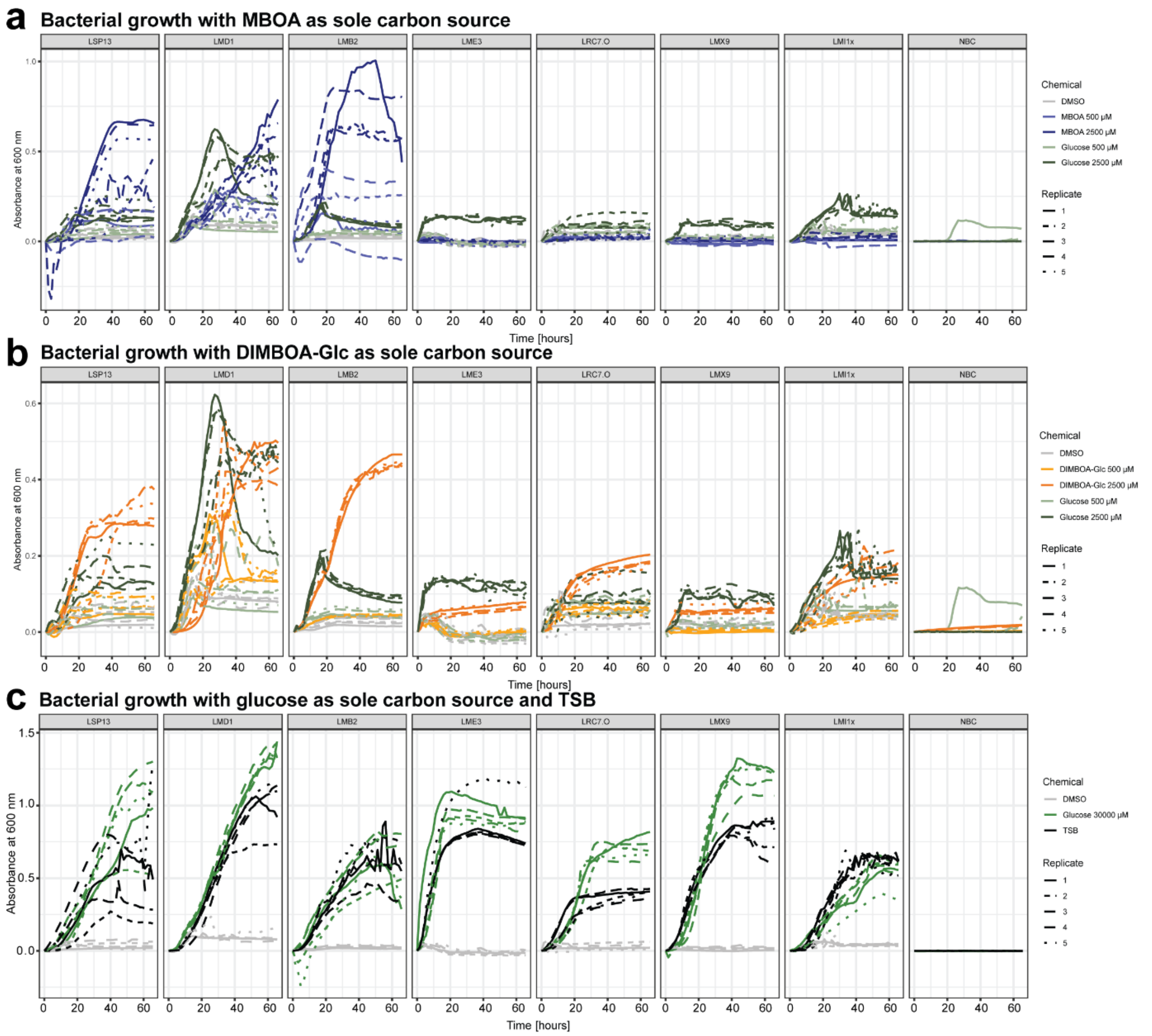

Supplementary Figure 6: Growth of maize root bacteria with benzoxazinoids as sole carbon source. a) Growth curves (absorbance at 600 nm) of bacteria growing in the minimal medium with the control treatment (DMSO), two concentrations of MBOA (500 µM and 2’500 µM) and the same concentrations of glucose as positive growth control. **b)** Same growth curves in the control treatment, two concentrations of DIMBOA-Glc (500 µM and 2’500 µM, note that the compound only has 70% purity) and with glucose. **c)** Growth curves in the control treatment and the positive control glucose (30000 µM) and complex medium 50% TSB (TSB). For each strain, five replicates were grown (n=5).

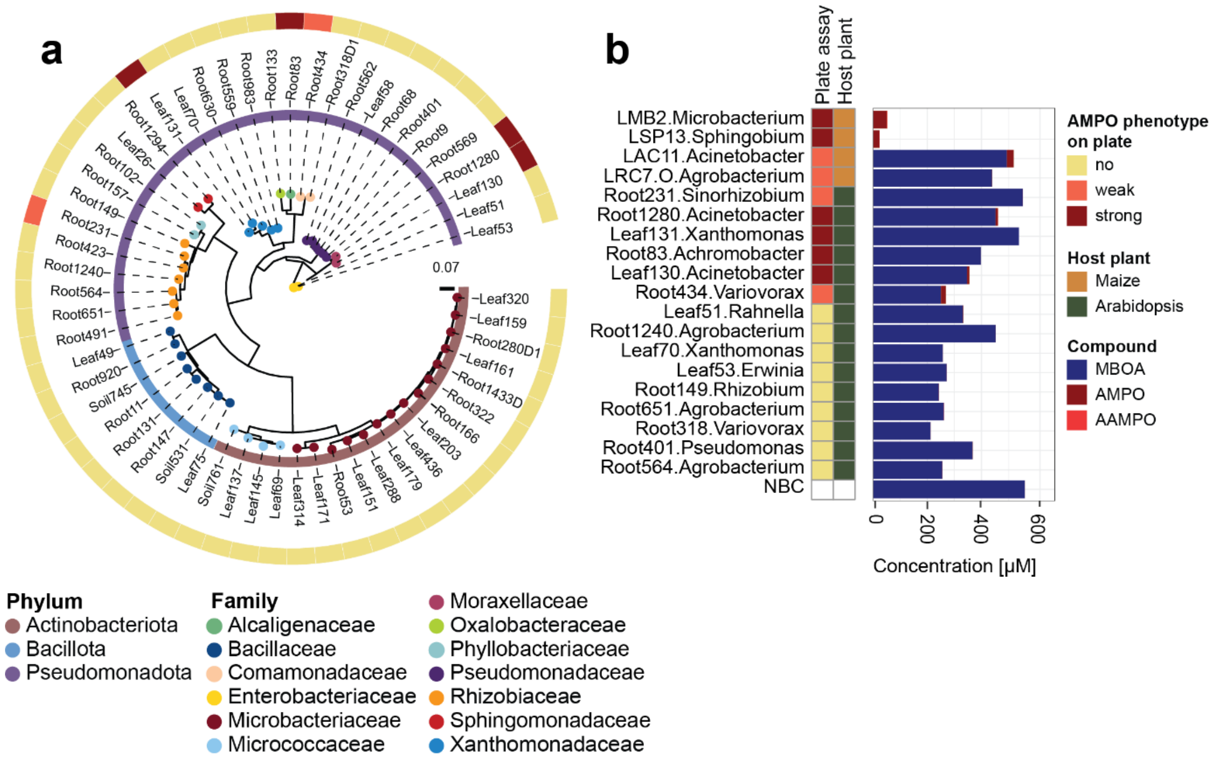

Supplementary Figure 7: Screening of Arabidopsis bacteria for AMPO formation a) Maximum likelihood phylogeny, constructed from the alignment of 16S rRNA gene sequences of Arabidopsis bacteria (AtSphere collection). Leaf nodes are coloured by family taxonomy and the ring next to the strain IDs reports phylum taxonomy. The surrounding ring displays the phenotype of the strains on MBOA containing plates and their phenotype classifications as strong, weak or non AMPO-formers. **b)** Quantitative MBOA metabolisation profiles of selected Arabidopsis bacteria. For this analysis, all strains with a colour change in the plate assay (**a**) were selected alongside some non AMPO-formers from similar taxa. As positive controls three MRB strains were included. All cultures were grown for 68 h in 50% TSB supplemented with 500 µM MBOA. The metabolite measurements (n=1) were made from three independently grown samples which were pooled in equal ratios before analysis.

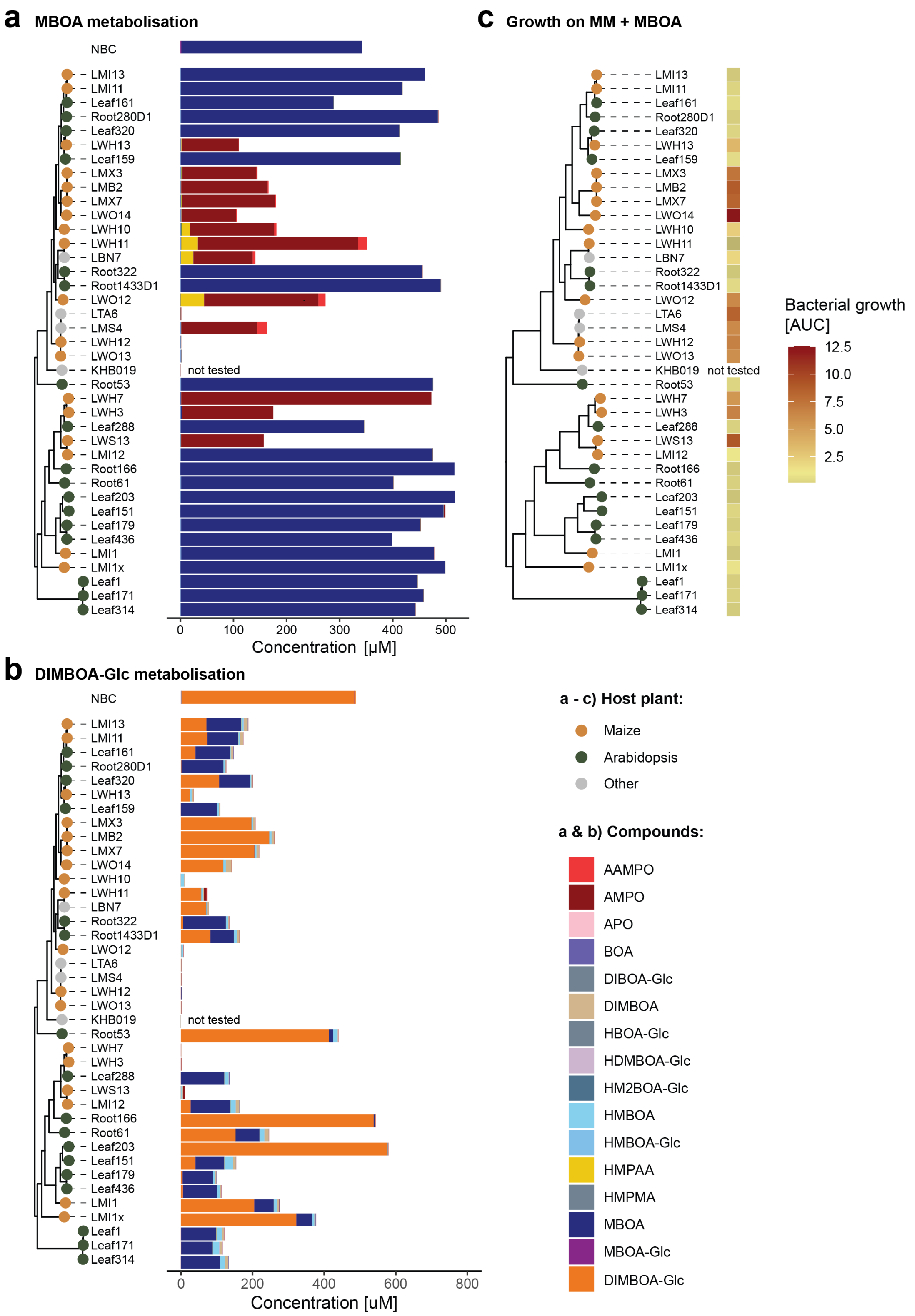

Supplementary Figure 8: Benzoxazinoid metabolisation by *Microbacterium* spp.: Phylogenetic tree with metabolite profiles of **a)** 500 µM MBOA and **b)** 500 µM DIMBOA-Glc as bar graphs. All cultures were grown for 68 h in liquid culture. The metabolite measurements (n=1) were made from three independently grown samples which were pooled in equal ratios before analysis. **c)** Total growth (representing area under growth curve over 68 h) in minimal medium with 500 µM MBOA as sole carbon source. Represented values are means from 12 independently grown cultures in two independent experiments. All cultures were grown for 68 h in liquid culture. This figure reports the quantitative data of Figure 3.

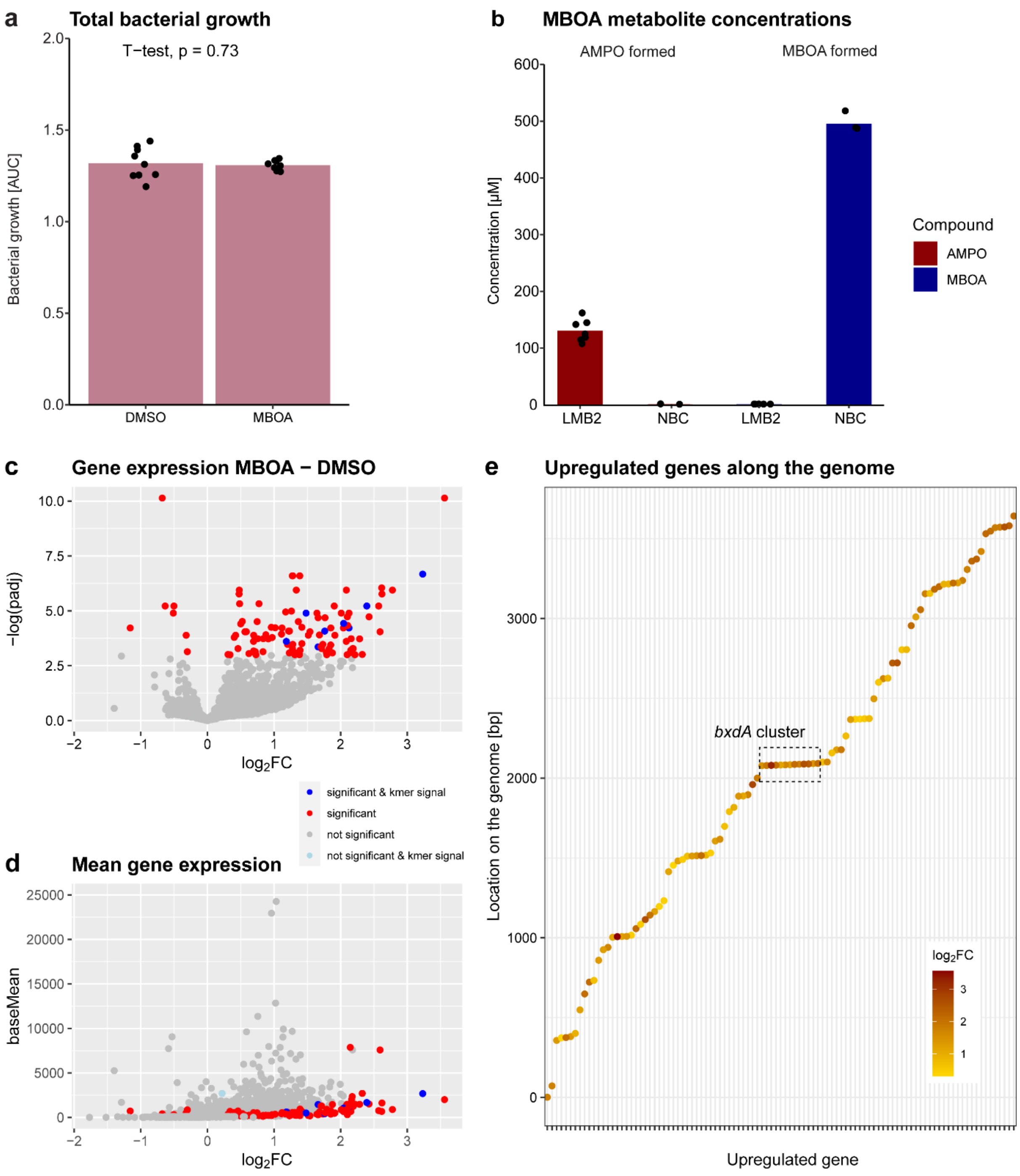

Supplementary Figure 9: Transcriptome analysis of *Microbacterium* LMB2 exposed to MBOA. *Microbacterium* LMB2 was grown in 50% TSB supplemented with DMSO or 500 µM MBOA for 16 h. **a)** Total growth of LMB2 assessed by absorbance at 600 nm. Shown is total growth (representing area under the curve, AUC) of 9 replicates each. **b)** Metabolisation profile of LMB2 (n = 7) and the negative control without bacteria (NBC, n= 3) grown in presence of MBOA. MBOA and AMPO are shown. **c)** Volcano plot and **d)** dot plot representing log_2-transformed fold-changes in response to MBOA and mean expression levels of differentially regulated genes (significant = adj. p-value < 0.05). Three very highly expressed (baseMean >25000) unregulated genes are not shown for simplicity. **e)** Dot plot showing significantly upregulated genes ($log_2FC > 0$) along the genome. The y-axis refers to the location on the genome (gene number), and the x-axis contains the upregulated genes sorted by gene number. The *bxd* gene cluster is highlighted with a black box.

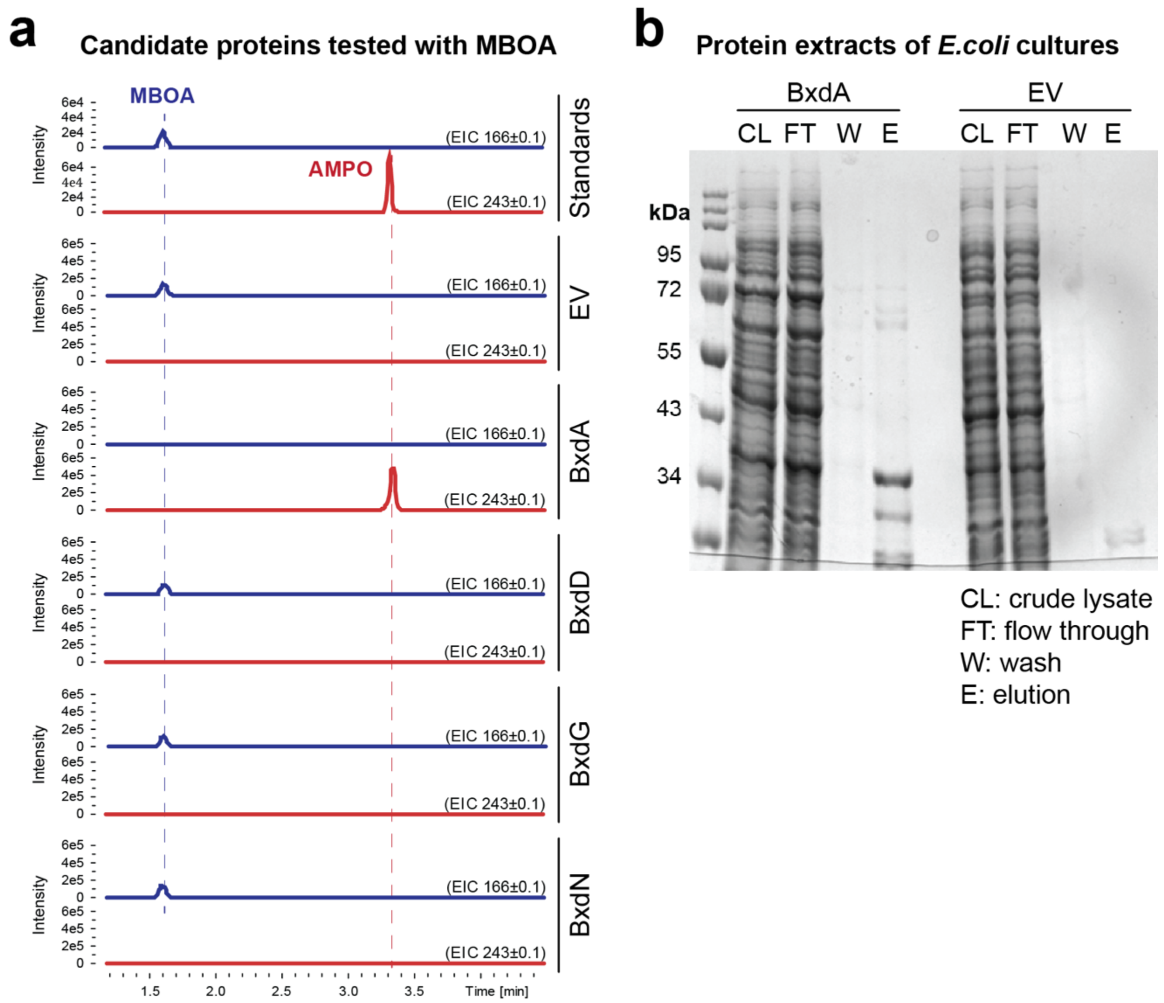

Supplementary Figure 10: BxdA converts MBOA to AMPO. a) Purified recombinant proteins of *E. coli* cultures (n=1) with empty vector (EV) as negative control or expressing BxdA, BxdD, BxdG or BxdN construct were incubated with the substrate MBOA, and product formation was monitored with high-pressure liquid chromatography-mass spectrometry (HPLC-MS) operated in positive mode (full-scan, EIC = extracted ion chromatogram). The EV control, BxdD, BxdG and BxdN showed no activity. Authentic MBOA and AMPO were used as standards. Note the different scales of the different y-axes. **b)** SDS-PAGE gel of protein (n=1) purification from *E. coli* cultures expressing *bxdA* (left) or the empty vector (EV, right) constructs.

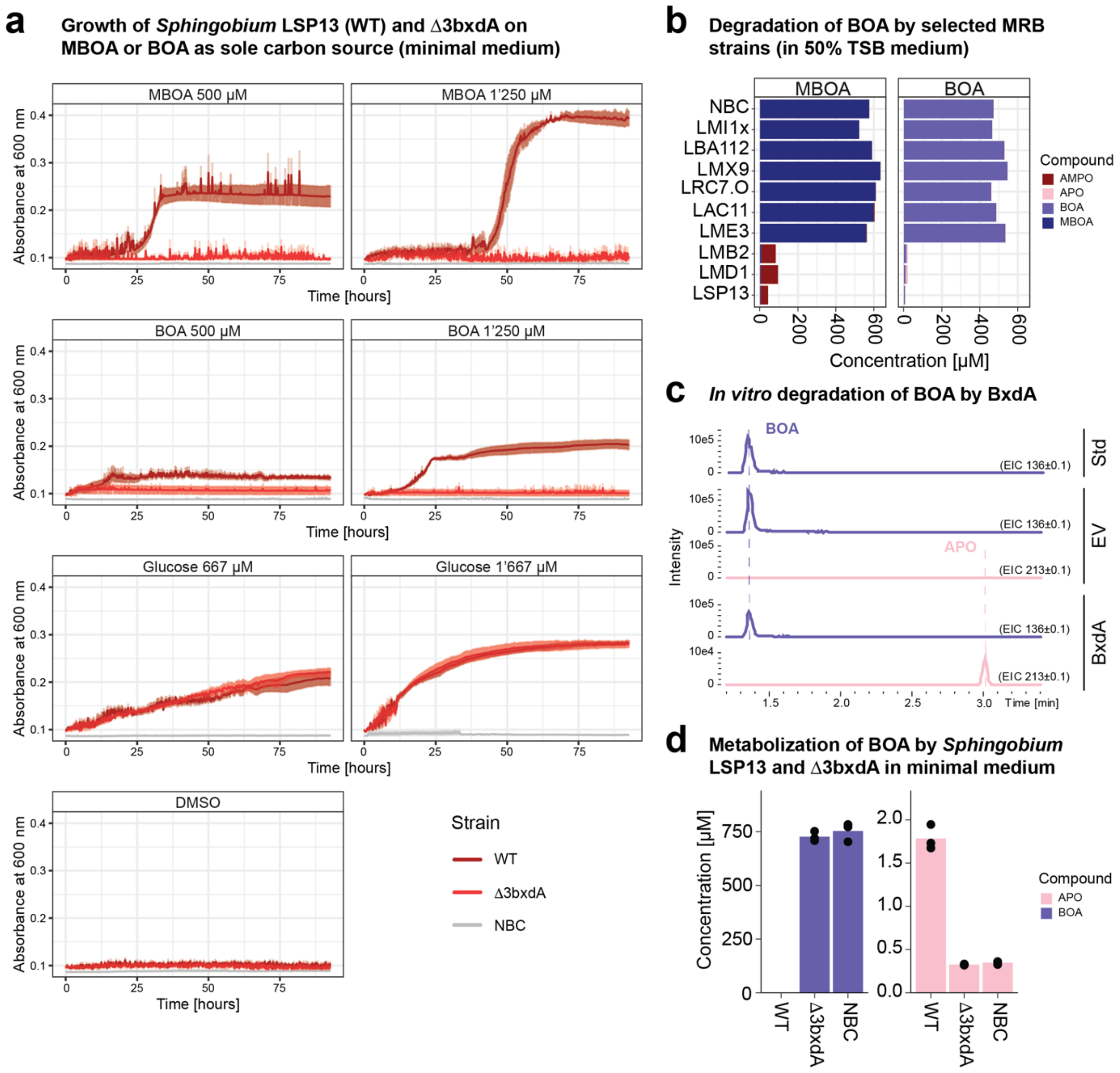

Supplementary Figure 11: Growth of the *Sphingobium* mutant Δ3bxdA with MBOA and BOA as sole carbon sources and additional experiments with BOA. a) Growth curves of *Sphingobium* LSP13 wild-type (WT) and the Δ3bxdA mutant in minimal medium containing MBOA or BOA as a sole carbon sources (500 and 1'250 µM). As a control, glucose was used at 667 µM and 1'667 µM corresponding to C equivalents of 500 µM MBOA and 1'250 µM MBOA, respectively. DMSO was supplied at 2 mg/L in all. Shown are the mean and SE of three biological replicates over 94 h. **b-d)** Complementary to the experiments with MBOA, we also tested the key observations with non-methoxylated BOA. **b)** Metabolisation profiles of selected strains (same set of strains as for kinetic characterisation of MBOA degradation and AMPO formation (Fig. 2c)) grown in 50% TSB supplemented with 500 µM MBOA or BOA. All cultures were grown for 68 h in liquid culture. The metabolite measurements were made from three independently grown samples which were pooled in equal ratios before analysis. **c)** In vitro degradation of BOA (1 mM starting concentration) by purified recombinant BxdA from LMB2 or empty vector control (EV). Note the different scales of the different y-axes. **d)** In vivo metabolisation of BOA by *Sphingobium* LSP13 WT and the Δ3bxdA mutant in minimal medium supplemented with 500 µM after 68 h. Shown are means and individual datapoints of 3 replicates.

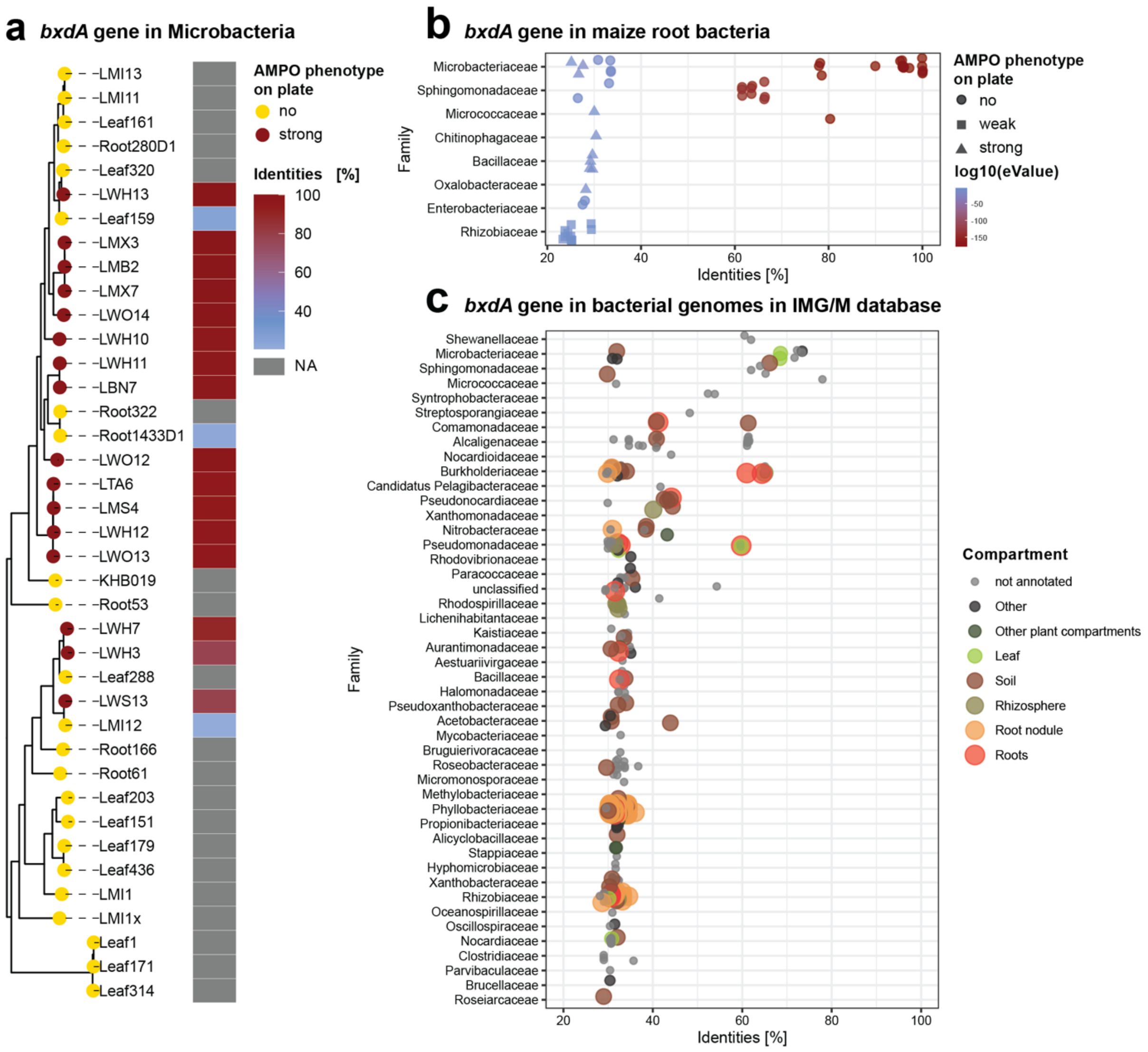

Supplementary Figure 12: BxdA is specific to maize bacteria. a) Homology searches with the protein BxdA of the *Microbacterium* strain LMB2 across all microbacteria used in this study, **b)** across all genome-sequenced strains of our MRB collection and **c)** against the Integrated microbial genomes / microbiome (IMG/M) database run by the Joint Genome Institute (JGI) using default parameters. BLASTP outputs report the % protein similarity (accessed December 2023).

Table S1: List of genes present in the *bxd* gene cluster and information on identification of association by different methods.

Gene	Locus Nr[1]	Annotation	Type	OG method[2]				Kmer[3]		RNAseq[4]	
				OG	sensitivity	specificity	adj. *P*	seqs	adj. *P*	$\log_2$FC	adj. *P*
bxdA	002078	N-acyl homoserine lactonase family protein	Enzyme	OG0002374	100	83	5.0E-05	**13**	**9.2E-10**	**1.5**	**7.5E-03**
bxdB	002079	RidA family protein	Enzyme	**OG0001787**	**100**	**100**	**8.6E-09**			**1.9**	**5.4E-03**
bxdC	002080	acyl-CoA dehydrogenase family protein	Enzyme	OG0000173	100	91	1.3E-06	**12**	**2.5E-07**	**3.2**	**1.3E-03**
bxdD	002081	aldehyde dehydrogenase family protein	Enzyme					**13**	**9.2E-10**	**2.0**	**1.2E-02**
bxdE	002082	thiamine pyrophosphate-dependent enzyme	Enzyme	**OG0002973**	**100**	**100**	**8.6E-09**	**13**	**9.2E-10**	**1.2**	**2.7E-02**
bxdF	002083	2-oxo acid dehydrogenase subunit E2	Enzyme	**OG0002972**	**100**	**100**	**8.6E-09**	**14**	**9.2E-10**	**1.7**	**3.5E-02**
bxdG	002084	VOC family protein	Enzyme	OG0002811	100	91	1.3E-06			**1.3**	**4.3E-02**
bxdH	002085	GntR family transcriptional regulator	Transcriptional regulator	OG0002086	100	78	3.2E-04			1.0	7.9E-02
bxdI	002086	acyl-CoA dehydrogenase family protein	Enzyme	OG0000173	100	91	1.3E-06	**14**	**9.2E-10**	**2.1**	**1.5E-02**
bxdJ	002087	flavin reductase	Enzyme					**13**	**9.2E-10**	**1.8**	**1.7E-02**
bxdK	002088	RidA family protein	Enzyme	**OG0001787**	**100**	**100**	**8.6E-09**			**2.6**	**2.4E-03**
bxdL	002089	M24 family metallopeptidase	Enzyme	**OG0002149**	**100**	**100**	**8.6E-09**	**14**	**9.2E-10**	**2.4**	**5.4E-03**
bxdM	002090	LacI family DNA-binding transcriptional regulator	Transcriptional regulator							1.1	7.4E-02
bxdN	002091	NAD(P)-dependent oxidoreductase	Enzyme	**OG0002971**	**100**	**100**	**8.6E-09**			**1.4**	**3.0E-02**
bxdO	002092	NADPH-dependent F420 reductase	Enzyme	OG0002551	100	87	8.3E-06			**2.1**	**2.6E-03**

[1] Locus numbers corresponding to the gene locus tags with "MRBLMB2_" omitted.
[2,3] Fisher's Exact tests, p-values adjusted for multiple comparisons using the Benjamini-Hochberg method.
[4] Wald tests, p-values adjusted for multiple comparisons using the Bonferroni method.
[2-4] Results for the different methods used to identify the *bxd* gene cluster (see methods for details). Significant results are indicated in bold.

Supplementary Table 2: List of primers used for heterologous expression of LMB2 candidate proteins

Name	Direction	Target	Sequence
**bxdA_pF_Fw**	forward	*bxdA*	AAGTTCTGTTTCAGGGCCCGATGAGTGAGCGTAAAACGGAT
**bxdA_pF_Rv**	reverse	*bxdA*	ATGGTCTAGAAAGCTTTACTAAGTTAACAAAATCCCGGC
**bxdD_pF_Fw**	forward	*bxdD*	AAGTTCTGTTTCAGGGCCCGATGGCCATAATGCGGTCCG
**bxdD_pF_Rv**	reverse	*bxdD*	ATGGTCTAGAAAGCTTTATTAGGCCACCCAGACAGT
**bxdG_pF_Fw**	forward	*bxdF*	AAGTTCTGTTTCAGGGCCCGATGGCTGACGCTGTACG
**bxdG_pF_Rv**	reverse	*bxdF*	ATGGTCTAGAAAGCTTTATTAGCGCTCCGGATGG
**bxdN_pF_Fw**	forward	*bxdN*	AAGTTCTGTTTCAGGGCCCGGTAACTACAGTAGGCTTCTTAG
**bxdN_pF_Rv**	reverse	*bxdN*	ATGGTCTAGAAAGCTTTATCAGGACTGGCGGCG
**pOPINF_Fw**	forward	vector	TAATACGACTCACTATAGGG
**pOPINF_Rv**	reverse	vector	TAGCCAGAAGTCAGATGCT

Supplementary Table 3: List of plasmids used for generation of the Δ3bxdA *Sphingobium* mutant

Name	Description	Reference
**pAK405**	pBR322 *oriV*, RP4 *oriT*, *lacZ'*, *nptII*, *rpsL1*	[5]
**pTETSIX**	pMB1 *oriV*, P_{tuf}-*mCherry*, *bla*, *tetA(C)*, RP4 *oriT*	[6]
**pAW02**	pAK405 carrying flanking regions for MRBLSP13_002921 knock-out	this study
**pAW07**	pTETSIX carrying flanking regions for MRBLSP13_002227 knock-out	this study
**pAW09**	pTETSIX carrying flanking regions for MRBLSP13_003006 knock-out	this study

Supplementary Table 4: List of oligos used for generation of the Δ3bxdA *Sphingobium* mutant

Oligo	Sequence (5'-3')[1]	Purpose[2]	Ref.
**pK18F**	ATGCTTCCGGCTCGTATGTTG	Sequencing pAK405 derivatives	[5]
**pK18R**	TAAGTTGGGTAACGCCAGG	Sequencing pAK405 derivatives	[5]
**red-2**	GTCTGACGCTCAGTGGAACG	Sequencing pTETSIX derivatives	[6]
**oKS1**	AGGAAGCTTTCCGCCTTGCAATGGTCG	Ampl. upstream region of	this study
**oKS2**	AGATCTAGAGATTGGGGTACTCATTGCAC	LSP13_2921 for pAW02	this study
**oKS3**	AGGTCTAGAATCATTCACACCTGAGGAGC	Ampl. downstream region	this study
**oKS4**	GAGGAATTCGCGATATAGCCCATCATCTC	of LSP13_2921 for pAW02	this study
**oKS13**	CGCCATGTTTGCTCGAGGA	Mutant screening	this study
**oKS14**	CTCGAACGGATTGACATCCG	LSP13_2921	this study
**oKS15**	GCGATCAAGCCGTCAATCC	Mutant screening	this study
**oKS16**	TAACACCGTCAGGCCACAC	LSP13_3006	this study
**oKS17**	CAACTGGGTGCCAGGTATAA	Mutant screening	this study
**oKS18**	AGCGATCGGTTCCGATCGT	LSP13_2921	this study
**oKS23**	ATATCTAGAAGGTCCGATCAGTCGCGA	Ampl. upstream region of	this study
**oKS24**	ATAGGTACCTATAATCGATGGGCCGACCAC	LSP13_2227 for pAW07	this study
**oKS25**	ATAGGTACCCCACACCTAAACGTACAGGC	Ampl. downstream region	this study
**oKS26**	ATAATGCATGCGCAACGAATATGATTGCG	of LSP13_2227 for pAW07	this study
**oKS27**	ATATCTAGAAAGGCCGGCAGCTACAAC	Ampl. upstream region of	this study
**oKS28**	ATAGGTACCGCCGAAAAAGTCCTGCAT	LSP13_3006 for pAW09	this study
**oKS29**	ATAGGTACCGCACGAAAGTCTACACCTGA	Ampl. downstream region	this study
**oKS30**	ATAATGCATCGCCGAAGAAGCAGGAAAAT	of LSP13_3006 for pAW09	this study
**oKS33**	CAGCACGAGACCGAAGAGGA	Mutant confirmation	this study
**oKS34**	GATCTGGTTGAACGCGTTGC	LSP13_2227	this study
**oKS35**	AGACCGAAGAGGAAAAGGCT	Mutant confirmation	this study
**oKS36**	GGAAGACGCTCAACTGGTTC	LSP13_2921	this study
**oKS37**	GTCGAAGCAATCCTCGTGAC	Mutant confirmation	this study
**oKS38**	CCGAAGGTGTGGCTTTCCTT	LSP13_3006	this study
**oKS40**	GGATGACGATGAGCGCATTG	Sequencing pTETSIX derivatives	this study
**oKS41**	GAAGGTTATCAGGGAACCCG	Mutant confirmation	this study
**oKS42**	GCGATGGCATGAGATAACCC	LSP13_2921	this study
**oKS43**	TTTTCAGGTGACGCCTGACC	Mutant confirmation	this study
**oKS44**	ATGCTTTCGCCTGCTCAAGG	LSP13_2227	this study
**oKS45**	GGTCTTTCCGCATCCTACTT	Mutant confirmation	this study
**oKS46**	CTGTTTACCGCTGGCCATTT	LSP13_3006	this study

[1] restriction sites are underlined

[2] "MRBLSP13_00" is for better readability abbreviated to "LSP13_" in gene names.

Supplementary References

1. Bai, Y. *et al.* Functional overlap of the Arabidopsis leaf and root microbiota. *Nature* **528**, 364–369 (2015).
2. Frey, M. *et al.* Benzoxazinoid biosynthesis, a model for evolution of secondary metabolic pathways in plants. *Phytochemistry* **70**, 1645–1651 (2009).
3. Hu, L. *et al.* Root exudate metabolites drive plant-soil feedbacks on growth and defense by shaping the rhizosphere microbiota. *Nature Communications* **9**, 2738–2738 (2018).
4. Cadot, S. *et al.* Specific and conserved patterns of microbiota-structuring by maize benzoxazinoids in the field. *Microbiome* **9**, 103–103 (2021).
5. Kaczmarczyk, A., Vorholt, J. A. & Francez-Charlot, A. Markerless gene deletion system for sphingomonads. *Applied and Environmental Microbiology* **78**, 3774–3777 (2012).
6. Ledermann, R., Strebel, S., Kampik, C. & Fischer, H.-M. Versatile Vectors for Efficient Mutagenesis of Bradyrhizobium diazoefficiens and Other Alphaproteobacteria. *Applied and Environmental Microbiology* **82**, 2791–2799 (2016).
