# Supplementary material for: The lactonase BxdA mediates metabolic specialisation of maize root bacteria to benzoxazinoids
Source: Nat Commun. 2024 Aug 2;15:6535. doi: 10.1038/s41467-024-49643-w (PMC11297187; doi:10.1038/s41467-024-49643-w)
Supplement: Supplementary file 3 — Description of Additional Supplementary Files [file 41467_2024_49643_MOESM3_ESM.pdf]

<u>Description of Additional Supplementary Files</u>

Supplementary Data 1: Table listing all bacterial strains used for this study including the three *Microbacterium* strains isolated and sequenced in this study, maize root bacteria (MRB) and Arabidopsis bacteria (AtSphere).

Supplementary Data 2: Excel file listing all the results of the OrthoFinder approach for all orthogroups across the genome across the microbacteria.

Supplementary Data 3: Table listing the kmers with the highest scores across the microbacteria.

Supplementary Data 4: The file reporting the expression, the differential change between the treatments and the statistics of all the genes in the *Microbacterium* LMB2 genome.

Supplementary Data 5: Excel file including the results of the blast of *bxdA* to the IMG database of JGI.
