# Supplementary material for: The lactonase BxdA mediates metabolic specialisation of maize root bacteria to benzoxazinoids
Source: Nat Commun. 2024 Aug 2;15:6535. doi: 10.1038/s41467-024-49643-w (PMC11297187; doi:10.1038/s41467-024-49643-w)
Supplement: Supplementary file 9 — Reporting Summary [file 41467_2024_49643_MOESM9_ESM.pdf]

nature portfolio

Corresponding author(s): Klaus Schlaeppi

Last updated by author(s): Jun 7, 2024

Reporting Summary

Nature Portfolio wishes to improve the reproducibility of the work that we publish. This form provides structure for consistency and transparency in reporting. For further information on Nature Portfolio policies, see our Editorial Policies and the Editorial Policy Checklist.

Statistics

For all statistical analyses, confirm that the following items are present in the figure legend, table legend, main text, or Methods section.

n/a	Confirmed	
☐	☒	The exact sample size (*n*) for each experimental group/condition, given as a discrete number and unit of measurement
☐	☒	A statement on whether measurements were taken from distinct samples or whether the same sample was measured repeatedly
☐	☒	The statistical test(s) used AND whether they are one- or two-sided<br>*Only common tests should be described solely by name; describe more complex techniques in the Methods section.*
☒	☐	A description of all covariates tested
☐	☒	A description of any assumptions or corrections, such as tests of normality and adjustment for multiple comparisons
☐	☒	A full description of the statistical parameters including central tendency (e.g. means) or other basic estimates (e.g. regression coefficient) AND variation (e.g. standard deviation) or associated estimates of uncertainty (e.g. confidence intervals)
☐	☒	For null hypothesis testing, the test statistic (e.g. *F*, *t*, *r*) with confidence intervals, effect sizes, degrees of freedom and *P* value noted<br>*Give P values as exact values whenever suitable.*
☒	☐	For Bayesian analysis, information on the choice of priors and Markov chain Monte Carlo settings
☒	☐	For hierarchical and complex designs, identification of the appropriate level for tests and full reporting of outcomes
☒	☐	Estimates of effect sizes (e.g. Cohen's *d*, Pearson's *r*), indicating how they were calculated

Our web collection on statistics for biologists contains articles on many of the points above.

Software and code

Policy information about availability of computer code

Data collection	We provide all source data (together with the analysis R scripts) on GitHub (https://github.com/PMI-Basel/Thoenen_et_al_AMPO_formation).
Data analysis	We analysed all our data in R and used R to prepare the manuscript figures. We provide all R scripts (together with the source data) on GitHub (https://github.com/PMI-Basel/Thoenen_et_al_AMPO_formation).

For manuscripts utilizing custom algorithms or software that are central to the research but not yet described in published literature, software must be made available to editors and reviewers. We strongly encourage code deposition in a community repository (e.g. GitHub). See the Nature Portfolio guidelines for submitting code & software for further information.

Data

Policy information about availability of data

All manuscripts must include a data availability statement. This statement should provide the following information, where applicable:

- Accession codes, unique identifiers, or web links for publicly available datasets
- A description of any restrictions on data availability
- For clinical datasets or third party data, please ensure that the statement adheres to our policy

The bacterial genome data (raw reads and annotated genomes) is available from the BioProjects PRJNA1009252 (MRB collection21) and PRJNA1083430 (new strains). We have deposited the sequences of the transcriptome experiment at NCBI's Gene Expression Omnibus (GEO) under the accession number GSE263275. We have deposited all source data together with all analysis scripts on GitHub.

Research involving human participants, their data, or biological material

Policy information about studies with human participants or human data. See also policy information about sex, gender (identity/presentation), and sexual orientation and race, ethnicity and racism.

Reporting on sex and gender	not applicable
Reporting on race, ethnicity, or other socially relevant groupings	not applicable
Population characteristics	not applicable
Recruitment	not applicable
Ethics oversight	not applicable

Note that full information on the approval of the study protocol must also be provided in the manuscript.

Field-specific reporting

Please select the one below that is the best fit for your research. If you are not sure, read the appropriate sections before making your selection.

☐ Life sciences ☐ Behavioural & social sciences ☒ Ecological, evolutionary & environmental sciences

For a reference copy of the document with all sections, see nature.com/documents/nr-reporting-summary-flat.pdf

Ecological, evolutionary & environmental sciences study design

All studies must disclose on these points even when the disclosure is negative.

Study description	We studied the metabolisation of benzoxazinoids (main compounds in the exudates of maize) by maize root bacteria.
Research sample	We have tested our culture collection of maize root bacteria for their ability to metabolize the main benzoxazinoid accumulating in the rhizosphere of maize. This was done using a visual screen (the metabolisaiton product AMPO is red) and also with analyitical methods. We compared the genomes of AMPO forming vs. non-forming Microbacteria strains to identify the genetic basis of AMPO formation. Sphingobium bacteria were also strong AMPO formers and we found the same genes in their genome. A mutant analysis then provide the in vivo prove that these bacteria also use BxdA to metabolise MBOA to AMPO.
Sampling strategy	We used independent methodological approaches to narrow down the candidate genes for AMPO formation.
Data collection	Different types of data were collected.: 1) Phenotypic data of the AMPO-formation on plate was recorded with images, eventually counted as nr of forming colonies and documented in spreadsheets. 2) analytical data was recorded from the UPLC-MS output. 3) bacterial growth based on OD600 measurementswas recorded in a plate reader and the growth curves analyzed in R. 4) genomes were generated from DNA of purified strains.
Timing and spatial scale	The timing depended on the assays. 10d for the plate assays, 68h for the liquid assay.
Data exclusions	For this study we started with an initial screening and with repeated validations, we narrowed down the selection of strains for comparative genomics and functional analyses. The strains that failed in repeated validations were 'left behind'.
Reproducibility	Our maxime to secure reproducibility is independent validation. Either we do this with a different approaches (plate and liquid assay; orthoggroups, kmer and transcriptome) or with replicated experiments.
Randomization	We used a partially randomized setup for the plate assays, where the bacteria were positioned at different positions in the plates in replicated assays.
Blinding	Blinding was hardly possible for this study, as we performed a screening of single bacteria without treatment groups. Also the genetic work (generation of mutant in LSP13) was done without blinding.

Did the study involve field work? ☐ Yes ☒ No

Reporting for specific materials, systems and methods

We require information from authors about some types of materials, experimental systems and methods used in many studies. Here, indicate whether each material, system or method listed is relevant to your study. If you are not sure if a list item applies to your research, read the appropriate section before selecting a response.

Materials & experimental systems

n/a	Involved in the study
☒	☐ Antibodies
☒	☐ Eukaryotic cell lines
☒	☐ Palaeontology and archaeology
☐	☒ Animals and other organisms
☒	☐ Clinical data
☒	☐ Dual use research of concern
☐	☒ Plants

Methods

n/a	Involved in the study
☒	☐ ChIP-seq
☒	☐ Flow cytometry
☒	☐ MRI-based neuroimaging

Animals and other research organisms

Policy information about studies involving animals; ARRIVE guidelines recommended for reporting animal research, and Sex and Gender in Research

Laboratory animals	This study involved bacteria isolated from maize roots. The taxonomies of all 110 strains is reported in Dataset S1.
Wild animals	These bacteria were mostly isolated from wild-type maize plants that were grown in natural soil. We have described the isolation effort of these bacteria in an earlier publication (Thoenen et al. 2023, PNAS)
Reporting on sex	not applicable
Field-collected samples	We have described the isolation effort of these bacteria in an earlier publication (Thoenen et al. 2023, PNAS)
Ethics oversight	No ethical approval was required for this study

Note that full information on the approval of the study protocol must also be provided in the manuscript.

Plants

Seed stocks	We grew wild-type maize plants (inbred line B73) and BX-deficient bx1(B73) maize, wheat (variety CH Claro), Medicago sativa (from Sativa, Rheinau, Switzerland), Brassica napus (Botanik Saemereien AG, Pfaeffikon, Switzerland) and Arabidopsis thaliana (Col-0)
Novel plant genotypes	This work did not generate novel plant genotypes
Authentication	Not applicable
